# Susceptibility to mycobacterial disease due to mutations in IL-12Rβ1 in three Iranian patients

**DOI:** 10.1007/s00251-017-1041-3

**Published:** 2017-12-18

**Authors:** Maryam Alinejad Dizaj, Esmaeil Mortaz, Seyed Alireza Mahdaviani, Davood Mansouri, Payam Mehrian, Els M. Verhard, Mohammad Varahram, Delara Babaie, Ian M. Adcock, Johan Garssen, Esther van de Vosse, Aliakbar Velayati

**Affiliations:** 1 Department of Biotechnology, School of Advanced Technologies in Medicine, Shahid Beheshti University of Medical Sciences, Tehran, Iran; 2 Chronic Respiratory Diseases Research Center, National Research Institute of Tuberculosis and Lung Diseases (NRITLD), Shahid Beheshti University of Medical Sciences, Tehran, Iran; 3 Clinical Tuberculosis and Epidemiology Research Center, National Research Institute of Tuberculosis and Lung Diseases (NRITLD), Shahid Beheshti University of Medical Sciences, Tehran, Iran; 4 Division of Pharmacology, Utrecht Institute for Pharmaceutical Sciences, Faculty of Science, Utrecht University, Utrecht, The Netherlands; 5 Pediatric Respiratory Diseases Research Center, National Research Institute of Tuberculosis and Lung Diseases (NRITLD), Shahid Beheshti University of Medical Sciences, Tehran, Iran; 6 Department of Infectious Diseases, Leiden University Medical Center, Leiden, The Netherlands; 7 Mycobacteriology Research Center, National Research Institute of Tuberculosis and Lung Diseases (NRITLD), Shahid Beheshti University of Medical Sciences, Tehran, Iran; 8 Department of Allergy and Clinical Immunology, Mofid Children’s Hospital, Shahid Beheshti University of Medical Sciences, Tehran, Iran; 9 Cell and Molecular Biology Group, Airways Disease Section, National Heart and Lung Institute, Imperial College London, Dovehouse Street, London, UK; 10 Priority Research Centre for Healthy Lungs, Hunter Medical Research Institute, The University of Newcastle, Newcastle, New South Wales Australia; 11 Nutricia Research Centre for Specialized Nutrition, Utrecht, The Netherlands

**Keywords:** IFN-γ, IL12RB1, IL-12Rβ1, IMD30, MSMD, PID

## Abstract

In the last decade, autosomal recessive interleukin-12 receptor β1 (IL-12Rβ1) deficiency, the most common cause of Mendelian susceptibility to mycobacterial disease (MSMD), has been diagnosed in a few children and adults with severe tuberculosis in Iran. Here, we report three cases referred to the Immunology, Asthma and Allergy ward at the National Research Institute of Tuberculosis and Lung Diseases (NRITLD) at Masih Daneshvari Hospital from 2012 to 2017 with Mycobacterium tuberculosis and non-tuberculous mycobacteria infections due to defects in IL-12Rβ1 but with different clinical manifestations. All three were homozygous for either an IL-12Rβ1 missense or nonsense mutation that caused the IL-12Rβ1 protein not to be expressed on the cell membrane and completely abolished the cellular response to recombinant IL-12. Our findings suggest that the presence of IL-12Rβ1 deficiency should be determined in children with mycobacterial infections at least in countries with a high prevalence of parental consanguinity and in areas endemic for TB like Iran.

## Introduction

Natural human immunity to intracellular pathogens, including opportunistic or low-virulence mycobacteria such as Bacillus Calmette-Guérin (BCG; an attenuated *Mycobacterium bovis* strain) vaccines, non-tuberculous mycobacteria (NTM) and non-typhoid Salmonella, and/or *Mycobacterium tuberculosis* (MTB), relies on the functional integrity of the interleukin (IL) 12/23-interferon (IFN)-γ axis enabling cross-talk between macrophages and T-lymphocytes/NK cells (Lee et al. 2011; Mortaz et al. 2015; Prando et al. 2013). Inborn errors of immunity (primary immunodeficiency) such as Mendelian susceptibility to mycobacterial disease (MSMD) emphasize the critical role of this axis in the defense against these pathogens (Casanova and Abel 2002; Feinberg et al. 2004; Haverkamp et al. 2014; Serour et al. 2007).

MSMD-causing mutations have been identified in eight autosomal genes (*IFNGR1*, *IFNGR2*, *STAT1*, *IL12B*, *IL12RB1*, *IRF8*, *ISG15*, and *TYK2*) and two X-linked genes (*IKBKG* and *CYBB*) (Bogunovic et al. 2012; Bustamante et al. 2011; Filipe-Santos et al. 2006; Hambleton et al. 2011; Haverkamp et al. 2014; Prando et al. 2013) resulting in impaired IFN-γ-mediated immunity (Casanova and Abel 2002; de Beaucoudrey et al. 2010).

IFN-γ is essential for killing and controlling mycobacterial infections (Casanova and Abel 2002; Lee et al. 2011). Complete deficiency of either of the two IFN-γ receptor chains (IFN-γR1 and IFN-γR2) or signal transducer and activator of transcription (STAT)-1 is associated with the development of disseminated infection in early childhood, has a very poor prognosis, and is often fatal. Complete IL-12p40 and IL-12Rβ1 deficiencies, as well as partial IFN-γR1, IFN-γR2, and *STAT1* deficiencies, are generally associated with a later onset disease, milder clinical infections, and a good prognosis (Altare et al. 2001; Casanova and Abel 2002; Casanova et al. 2002; Ottenhoff et al. 2002; Özbek et al. 2005; Reichenbach et al. 2001).

Genetic analysis of a large cohort of 220 MSMD patients with a defective IL-12/IL-23-IFN-γ axis ranked the presence of these mutations among MSMD patients as *IL12RB1* (40%), *IFNGR1* (39%), *IL12B* (9%), *STAT1* (5%), *IFNGR2* (4%), and *IKBKG* (3%) (Casanova et al. 1995; Filipe-Santos et al. 2006; Lee et al. 2011). In addition, IL-12Rβ1 deficiency was the first primary immunodeficiency to be associated with pediatric tuberculosis in children with normal resistance to BCG and environmental mycobacteria (EM) (de Beaucoudrey et al. 2010). IL-12Rβ1 deficiency, therefore, should probably be suspected in any patient with an unusual infection with intracellular pathogens even in the absence of parental consanguinity.

IL-12Rβ1 deficiency has been diagnosed in several children and teenagers with tuberculosis (TB). The prevalence of TB in IL-12p40- and IL-12Rβ1-deficient patients is however lower than BCG or NTM infection (Caragol et al. 2003; de Beaucoudrey et al. 2010; Özbek et al. 2005). Until 2005, only 4/73 (5.5%) patients with IL-12Rβ1 or IL-12p40 deficiency had been reported to have TB (3/54 patients with complete IL-12Rβ1 deficiency and 1/19 patients with complete IL-12p40 deficiency) (Fieschi and Casanova 2003; Özbek et al. 2005). BCG vaccination, and especially developing BCG disease, confers a greater protection against EM disease than against TB in IL-12Rβ1-deficient patients despite the close phylogenetic relationship between BCG and MTB, presumably because MTB is more virulent than EM (de Beaucoudrey et al. 2010).

Disseminated disease in children and pulmonary disease in adults constitute two major epidemiological and clinical forms of TB infection (Alcaïs et al. 2005). Understanding the causes of mycobacterial infection in children and adults and finding the driver mutations in the IL-12/IL-23-IFN-γ axis may provide a new approach for controlling the tuberculosis infections. Here, we report three MSMD patients from Iran with MTB and NTM infection due to a complete inability to produce IFN-γ in response to IL-12 due to mutations in IL-12Rβ1.

## Case reports

The three children were either born to consanguineous parents (patients 1 and 3) or to unrelated parents (patient 2) and were referred to the Immunology, Asthma and Allergy ward at the National Research Institute of Tuberculosis and Lung Diseases (NRITLD) at Masih Daneshvari Hospitals, Shahid Beheshti University of Medical Sciences, Tehran-Iran, between January 2012 and July 2017.

## Patient 1

**Fig. 1:**
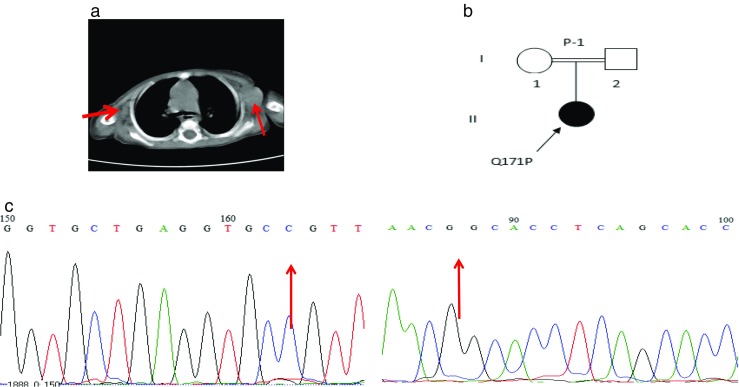
Patient 1. **a** CT scan without contrast. Note bilateral axillary adenopathies (red arrow). **b** Pedigree of patient 1. **c** Sequencing analysis by dideoxy-nucleotide termination method demonstrated a mutation in exon 5 of the *IL-12RB1* gene using forward (left panel) and reverse (right panel) sequencing

This was an 8-year-old female born to consanguineous parents from the center of Iran (Table 1; patient 1). She was vaccinated with live attenuated *Mycobacterium bovis* BCG sub-strain Pasteur at birth. Two months later, she developed a progressive bilateral axillary adenopathy (Fig. 1a) but was discharged without any medical intervention. At the age of 6 months, the patient developed supraclavicular lymphadenopathy. MTB complex was detected in drainage secretions and anti-TB medication was initiated and continued for 6 months.

**Table 1:** Genotypes and clinical phenotypes of patients with IL-12Rβ1 deficiency

Patient no.	Age^a^/sex	Age of onset (months)	Kind of mycobacterial infection	Clinical manifestations after BCG vaccination	Involved exon/mutation
1	8.3/F	3	BCG	Bilateral axillary adenopathy	Exon 5/c.512A>C, Q171P
2	3.7/M	3	BCG	Left axillary lymphadenopathy	Exon 9/c.847C>T, R283X
3	7.3/M	5	BCG and MTB	Axillary lymphadenopathy	Exon 5/c.517C>T, R173W

*BCG* Bacille-Calmette-Guérin, *MTB Mycobacterium tuberculosis*

^a^Age at death, last follow-up, or at the time of writing this report

Two years later, the patient was hospitalized due to high blood pressure, pyrexia, myalgia, cough, and severe weight loss. Evaluation of gastric washing (GW) cultures, smear-detected Mycobacterium, acid fast bacilli (AFB), and a polymerase chain reaction (PCR) test was positive for the MTB complex and BCG. Chest radiography showed right-sided pleural effusion and adenopathy in the para-aortic, celiac, and mesenteric regions was seen. Both kidneys were normal in size although evidence of abscesses on the left side was observed by ultrasound. PCR tests for urine and pleural fluid were also positive for BCG. An anti-TB treatment course was started with isoniazid, rifampin, ethambutol, Klacid, ofloxacin, and Lanoxin. Two months later, despite a negative PCR result for the MTB complex in urine and GW, the previous treatment regimen was complemented with clarithromycin, captopril, and IFN-γ (25 μg/m^2^). A few months later, AFB was isolated following bone marrow (BM) aspiration and an immunodeficiency was suspected.

Eight months later, clinical manifestations (persistent cough and dyspnea) continued to be reported despite treatment and the patient was admitted to the hospital. Various mycobacteriologic analyses (including sputum smears and cultures, drug susceptibility test (DST), PCR, and other tests) were performed. GW PCR showed the presence of MTB complex that was isoniazid- and rifampin-resistant. Blood tests showed a WBC of 6.1 × 10^3^/μl (Neu 71%; Lymph 22%; Mix 7%); Hb10.6 g/dl; HCT33%; and the ESR was 39 mm/h in the first hours with detected high C-reactive protein (CRP) (78 mg/l; normal range < 10 mg/l). Liver function tests revealed an aspartate aminotransferase (AST) of 36 u/l (normal 15–50 u/l), alanine aminotransferase (ALT) of 10 u/l (normal 10–25 u/l), and a decreased ALT/AST ratio. Antibody responses to tetanus and diphtheria were normal and DHR123 and nitroblue-tetrazolium (NBT) tests were within the normal range. The patient was treated again with amikacin, Klacid, cycloserine, Lanoxin, captopril, and IFN-γ (25 μg/m^2^). After a few months of therapy, whole blood was collected from both the patient and her parents and the release of IL-12 and IFN-γ from stimulated peripheral blood mononuclear cells (PBMCs) was assessed. In brief, PBMCs were isolated from whole blood containing EDTA using Ficoll-Paque before being stimulated with rhIL-12 (Gibco, Life Technologies, USA; 20 ng/ml), rhIFN-γ (Gibco, Life Technologies, USA; 5000 UI/ml), and/or BCG for 48 h and IFN-γ and IL-12 release was measured by ELISA.

IFN-γ release following stimulation with BCG + rhIL-12 was extremely low and a 100-fold lower than that observed from PBMCs from control subjects. Although the addition of rhIFN-γ to BCG-stimulated cells further increased IL-12p70 release from PBMCs 4.4-fold, this remained far less than that seen from normal healthy controls (Table 2; patient 1) and was similar to that reported in various MSMD patients (Feinberg et al. 2004). These functional data indicated a possible genetic defect in IL-12Rβ1.

**Table 2:** In vitro production of IFN-γ and IL-12p70 following stimulation in three IL-12Rβ1-deficient patients and healthy control subjects

Cytokine detected	Stimulus^a^	Patient 1	Patient 2	Patient 3	Healthy controls Mean ± SEM *n* = 6
IFN-γ (pg/ml)	Medium	0	0	1.8	37.4 ± 12.4
	BCG	47.2	15.4	35.04	1059.9 ± 136.9
	BCG + rhIL-12	80.24	16.9	42.04	16,703 ± 2036
	SI	1.7	1.1	1.2	16.6 ± 2.4
IL-12p70 (pg/ml)	Medium	0.9	17.1	0.16	2.7 ± 0.8
	BCG	49.8	64.8	77.5	75.8 ± 7.8
	BCG + rhIFN-γ	219.1	272.1	410.7	700.1 ± 89.9
	SI	4.4	4.2	5.3	9.5 ± 1.2

^a^Stimulation status: unstimulated (medium), stimulated with BCG alone, stimulated with BCG plus recombinant IL-12p70 (20 ng/ml), or stimulated with BCG plus recombinant IFN-γ (5000 UI/ml)

*SI* stimulation index (BCG + cytokine/BCG), *SEM* standard error of the mean

To examine this, genomic DNA was isolated from PBMCs by salt extraction and the IL-12Rβ1 coding exons and flanking regions of the introns were amplified by PCR and sequenced at Leiden University Medical Center and a homozygous mutation in *IL12RB1* exon 5, c.512A>C, which results in the amino acid substitution p.Q171P was identified (Fig. 1c). Both parents were heterozygous for this mutation. This mutation has been reported previously to result in complete lack of IL-12Rβ1 protein expression on the cell membrane (Fieschi et al. 2003). To our knowledge, this is the first case from Iran with this mutation.

## Patient 2

**Fig. 2:**
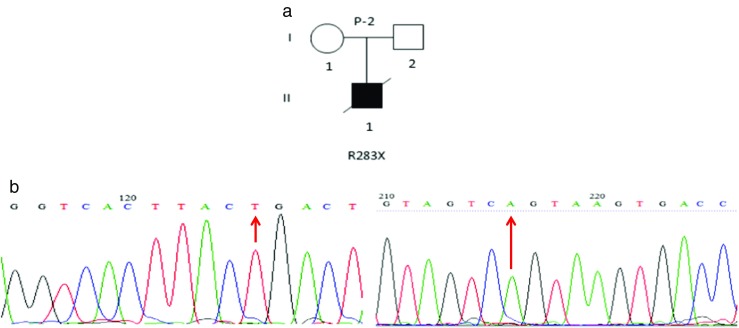
Patient 2. **a** Pedigree of patient 2. **b** Sequencing analysis by dideoxy-nucleotide termination method. A mutation in exon 9 of the *IL12RB1* gene was found in this patient; left and right panels indicate results for forward and reverse sequencing

The second case, a male born to unrelated parents and vaccinated with BCG at birth, was diagnosed with left axillary lymphadenopathy at the age of 3 months (Table 1; patient 2). Three months later, the patient developed bilateral inguinal lymphadenopathy. This was drained and the patient discharged. The patient was diagnosed with BCG-osis and treated with anti-TB drugs (rifampin, isoniazid, cycloserin, IFN-γ and, ethambutol), but despite this treatment, the patient was re-admitted to the hospital at the age of 7 months complaining of respiratory distress due to isoniazid use. Histological analysis of a cervical lymph node biopsy revealed proliferating activated histiocytes and macrophages, inflammatory cell infiltration, and a few scattered giant cells. PCR analysis of the lymph node biopsy was positive for the MTB complex and BCG but negative for viral infections including EBV and CMV. On physical examination, the patient had hepatosplenomegaly, lymphadenopathy in the para-aortic region and mesenteric roots, and bilateral axillary adenopathy. Treatment was continued for nearly 2 years but the patient’s general condition continued to deteriorate despite anti-TB treatment and he was again admitted to the hospital complaining of severe backache and abdominal ascites. Blood analysis gave the following results: ESR 11 mm/h, WBC 4.1 × 10^3^/μl (Neu 39.6%; Lymph 43.7%; Mix 16.7%), Hb 11.9 g/dl, PLT 120 × 10^3^/μl with a low liver function test ALT/AST ratio (310/345), and high alkaline phosphatase (ALP) 3400 u/ml (normal range 180–1200 u/ml). A qualitative C-reactive protein test was positive. An evaluation of the abdominal cavity by imaging and by physical examination showed moderate abdominal ascites and splenomegaly (116 mm). Liver biopsy showed chronic hepatitis, grade 3/18, stage 2/6. This condition is seen in both viral and autoimmune hepatitises. Plasma cell infiltration, lymphoid aggregates, or follicles (seen in autoimmune hepatitis) were absent and both liver biopsy and ascites were negative for AFB and mycobacteria. An MSMD functional assay was conducted in whole blood (PBMCs) and revealed a lack of response to recombinant IL-12 following BCG stimulation (Table 2; patient 2). Genomic sequence analysis revealed a c.847C > T mutation in exon 9 of the *IL12RB1* gene (Fig. 2b). This defect results in a change from R283 to a premature stop codon (p.R283X). Both parents were heterozygous for this mutation. This variation has been previously reported in a single patient from the Faroe Islands (Schejbel et al. 2011) and in two patients from Brazil (Falcão et al. 2012). Despite anti-TB and broad-spectrum antibiotic treatment, the patient’s general condition continued to deteriorate and the patient died a year after the detection of his immunodeficiency status at the age of 3 years and 7 months.

## Patient 3

The third patient was a 7-year-old male vaccinated with BCG at birth that developed axillary lymphadenopathy at 5 months of age (Table 1; patient 3). The family had no history of TB or exposure to MTB. The patient was referred to the hospital due to an abscess in the thoracic cavity at 15 months associated with high fever, cough, and anemia and with abdominal ascites. Whole blood analysis gave the following results: WBC 3.9 × 10^3^/μl (Neu 0%; Lymph 48%; Mono 49%; Mix 3%), Hb 6.3 g/dl, platelets 95 × 10^3^/μl, ESR 135 mm/h, and CRP 24 mg/lit. BM aspiration was hypocellular. An evaluation of the abdominal cavity by imaging and by physical examination showed abdominal ascites, splenomegaly, and lymphadenopathy in the para-aortic, mesenteric, and celiac regions. A tuberculin skin test was negative and serum levels of IgG and IgE were elevated. GW was positive for mycobacteria on Lowenstein-Jensen medium. The lymphocyte transformation test (LTT) for BCG was 2.1 (normal levels ≥ 2.5) and an NBT test was normal. Based on the physical analysis, laboratory data, and clinical signs, the patient underwent MSMD functional evaluation. A year and a half later, despite treatment with rifampin, isoniazid, ethambutol, and clarithromycin, the patient was re-admitted to the hospital due to backache, abdomen distension, and ascites. Radiologic analysis showed splenomegaly (126 mm). The ascites smear test was negative for AFB.

**Fig. 3:**
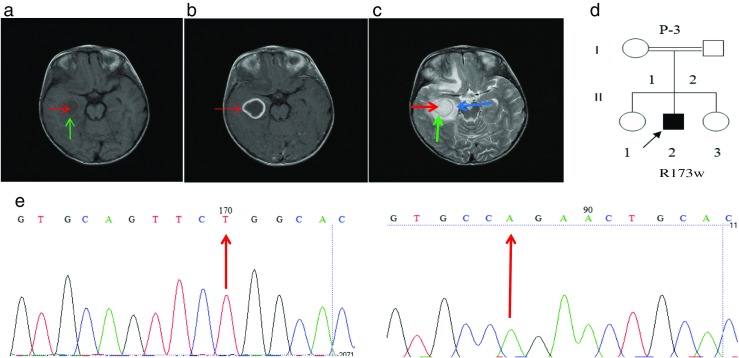
Patient 3. Cerebral abscesses. **a** Axial T1-weighted spin MRI image without contrast. There is a hypo-intense lesion (red arrow) with an iso-intense capsule (green arrow) posterior to temporal horn of the right lateral ventricle. The lesion has compressed the temporal horn. **b** Axial T1-weighted spine MRI image with contrast. The capsule demonstrates marked enhancement (red arrow). **c** Axial T2-weighted spine MRI image. The abscess is hyper-intense (red arrow). The capsule is iso-intense (green arrow) and the vasogenic edema surrounding the lesion (blue arrow) is also hyper-intense. **d** Pedigree of patient 3. **e** Sequencing analysis by dideoxy-nucleotide termination method demonstrated a mutation in exon 5 of the *IL12RB1* gene using forward (left panel) and reverse (right panel) sequencing

Four months later, the blood culture was positive for Kingella species and blood tests revealed the following results: WBC 7.05 × 10^3^/μl (Neu 72.3%; Lymph 10.1%; Mono 11.9%; Eos 5.4%; Baso 0.3%), Hb 12.4 g/dl, and PLT 241 × 10^3^/μl and the CD4/CD8 ratio was decreased compared to control levels (9/40). Serological assays and PCR for HIV were both negative. Thus, levofloxacin and IFN-γ were added to the treatment regimen (rifampin, isoniazid, ethambutol, amikacin). However, at 5.5 years of age, the general condition of the patient deteriorated severely and the patient was again admitted to the hospital complaining of seizure and experienced respiratory distress, scrotal edema, severe backache, abdominal ascites, and distension during the hospitalization. Complete blood analysis gave the following results: WBC 6.8 × 10^3^/μl (Neu 79%; Lymph 13%; Mix 8%), Hb 10.7 g/dl, and PLT 142 × 10^3^/μl and CRP was 61 mg/l. A low liver function test ALT/AST ratio (15/20) was observed. Multiple brain abscesses and cerebritis were seen by MRI (Fig. 3a–c), probably due to BCG-osis, and peripheral edema in the cerebrium was observed by computed tomography. The BACTEC test result for ascites and TB PCR for synovial fluid were all negative for AFB and TB. One year later, the brain abscesses were PCR-positive for TB that was rifampin-resistant.

Progression from localized disease into disseminated infection suggested an underlying immunodeficiency and a genetic and functional analysis of the IL-12/IL-23/IFN-γ loop was performed. Functional analysis revealed possible defects in the IL-12Rβ1 (Table 2; patient 3) and genetic testing revealed the homozygous *IL12RB1* mutation c.517C>T in exon 5 (Fig. 3e). This variation leads to an R173W amino acid change. Both parents of the patient were found to be heterozygous for this mutation. The R173W mutation has been previously reported in patients from Brazil, Venezuela, Poland (de Beaucoudrey et al. 2010), and Iran (Boisson-Dupuis et al. 2011). The previously reported Iranian patient had a history of PTB and cutaneous leishmaniasis (Boisson-Dupuis et al. 2011). To our knowledge, this is only the second patient in Iran reported with this mutation.

## Discussion

In young children, TB is often disseminated due to early, hematogenous spread of the Mycobacterium after primary pulmonary infection (Alcaïs et al. 2005). Clinical TB has now been described in a number of patients with IL-12/IL-23/IFN-γ system defects (Ottenhoff et al. 2005) and may result from autosomal recessive IL-12Rβ1 deficiency, in at least some children (Boisson-Dupuis et al. 2011). The present study reports three Iranian children with complete IL-12Rβ1 deficiency confirming previous studies in Iranian patients with an IL-12Rβ1 defect (Azam Sarrafzadeh et al. 2016; Boisson-Dupuis et al. 2011; de Beaucoudrey et al. 2010; Fieschi et al. 2003; Parvaneh et al. 2015; Tabarsi et al. 2011).

In the present study, we screened for IL-12Rβ1 mutations in suspected patients with MTB infection and reported three homozygous mutations: Q171P, R283X, and R173W. Patients 1 and 2 are the first reports of children with Mycobacterium infection due to IL-12Rβ1 deficiency in Iran. Patient 3, with an R173W mutation, is only the second child reported in Iran with this particular mutation (Boisson-Dupuis et al. 2011) and MTB infection. Interestingly, all three of the IL-12Rβ1-deficient patients reported here had been vaccinated with BCG and had developed BCG disease.

However, this deficiency is probably not unique to children, as a young adult case with TB and IL-12Rβ1 deficiency has also been identified in Iran (Tabarsi et al. 2011). The increasing reports of Iranian patients with defects in the IL-12/IL-23/IFN-γ axis (Azam Sarrafzadeh et al. 2016; Boisson-Dupuis et al. 2011; de Beaucoudrey et al. 2010; Mansouri et al. 2005; Parvaneh et al. 2015), particularly in areas with a high prevalence of parental consanguinity and a very low prevalence of HIV (less than 0.15%) (Boisson-Dupuis et al. 2011), suggest that screening for defects in this pathway should be considered in all cases. Thus, it will be important to define the prevalence of IL-12Rβ1 deficiency and related disorders among children and adults with severe TB. This is especially important in countries where the disease is endemic such as Iran (Tabarsi et al. 2011).

Mandatory screening for IL-12Rβ1 mutations that cause a defective IL-12/IL-23/IFN-γ axis would not only improve the quality of patient care but also increase the safety monitoring for potential complications including infectious diseases in these subjects. A global registry of patients with genetic defects in the IL-12/IL-23/IFN-γ axis such as that available at www.lovd.nl/IL12RB1 for IL-12Rβ1 defects, and that are also available for most other genes that cause MSMD, together with awareness of these immunodeficiencies prior to BCG vaccination will improve the health and quality of care for these patients.
